# Supplementary material for: Molecular recognition of the native HIV-1 MPER revealed by STED microscopy of single virions
Source: Nat Commun. 2019 Jan 8;10:78. doi: 10.1038/s41467-018-07962-9 (PMC6325134; doi:10.1038/s41467-018-07962-9)
Supplement: Supplementary file 1 — Supplementary Information [file 41467_2018_7962_MOESM1_ESM.pdf]

Supplementary Information

Molecular recognition of the native HIV-1 MPER revealed by STED microscopy of single virions

Carravilla et al.

Supplementary Figure 1

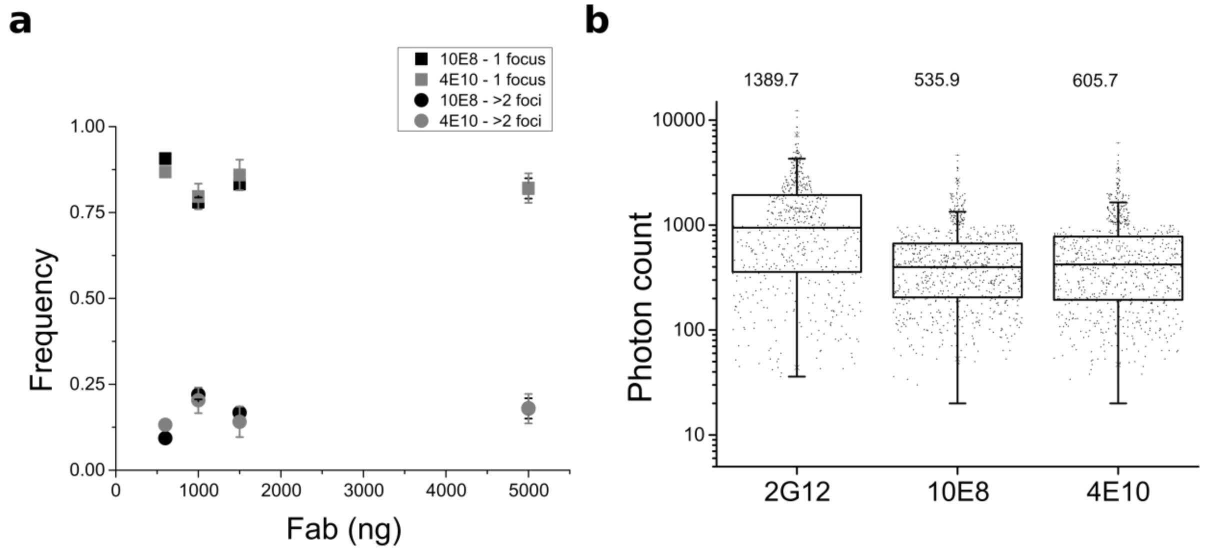

Supplementary Figure 1: Patterns of anti-MPER Fabs 4E10/10E8 binding to NL4-3 virions. (**a**) Env foci distribution detected by the labelled Fabs is shown at different Ab concentrations. Frequencies for 1 focus or >2 foci are displayed by the solid squares or circles, respectively. Error bars are SD of three independent experiments. (**b**) Median number of anti-human Fab-KK114 photons per viral particle reflects higher binding for 2G12 (n=578) as compared with 10E8 (n=753) and 4E10 (n=744) as revealed by secondary antibody. Only virions where antibodies were detected are plotted. Boxes are IQR and whiskers are SD.

Supplementary Figure 2

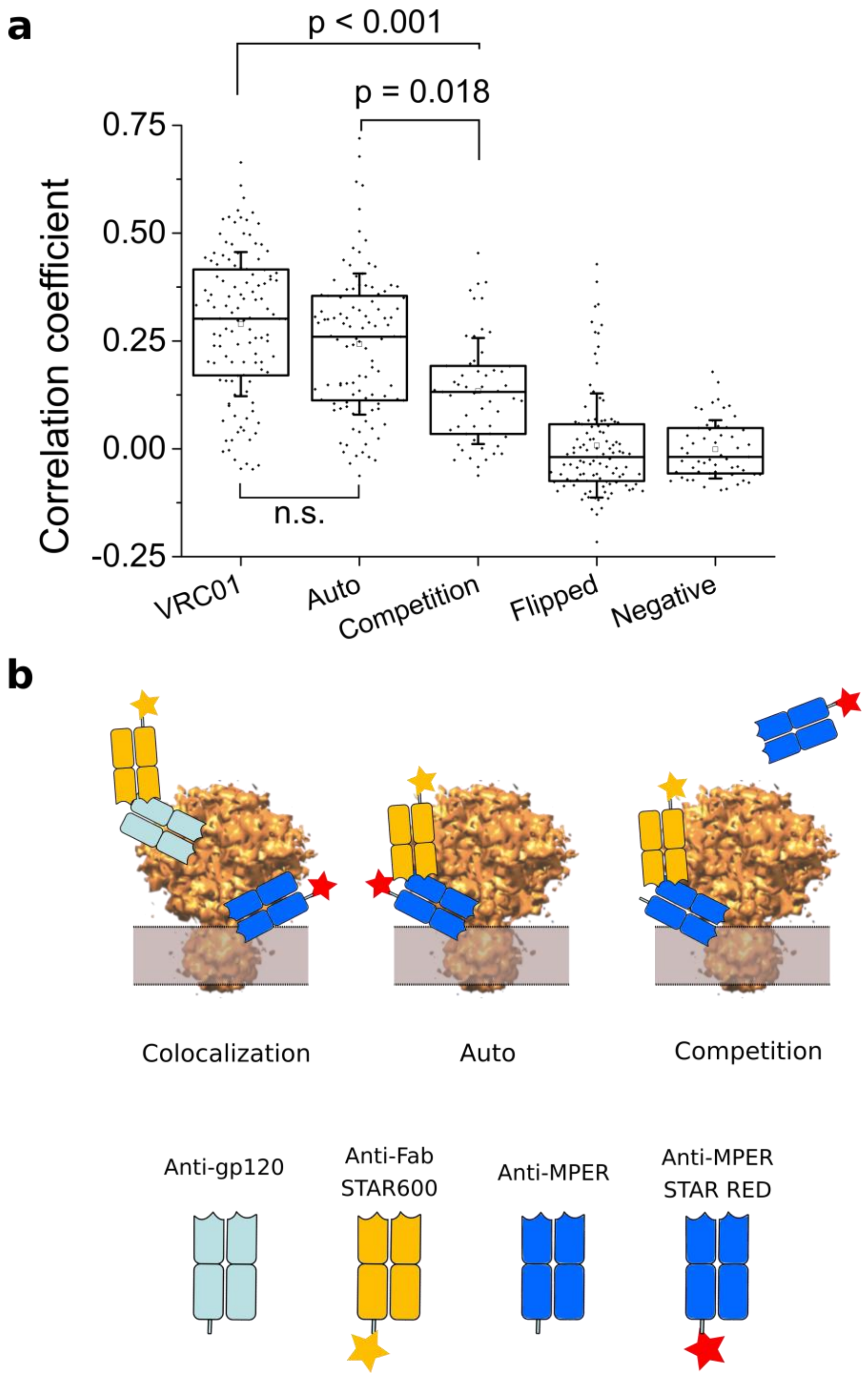

Supplementary Figure 2: Colocalization experiment formats. Pixel-wise linear correlation cefficients (**a**) measured for the colocalization of 10E8-KK114 (STAR RED) with anti-gp120 VRC01 (n=120), itself (Auto) (n=107), or 10E8-WT (Competition) (n=55), as depicted in the schematic diagrams in (**b**). Boxes are IQR and error bars SD. Statistical significance was assessed by Kruskal-Wallis test.

Supplementary Figure 3

a

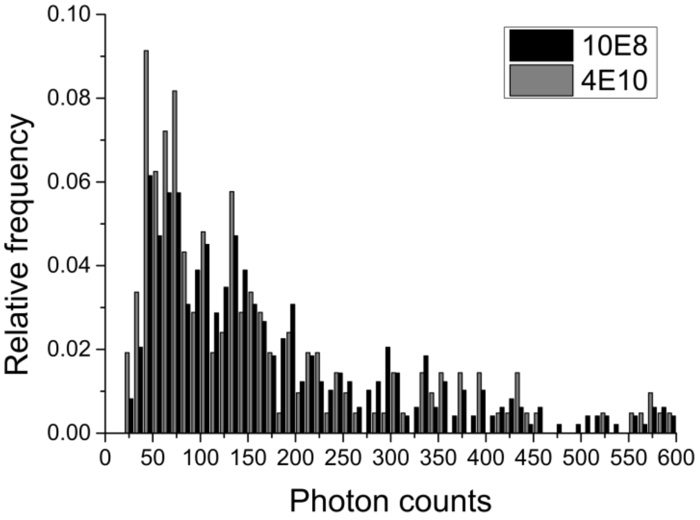

b

	10E8		4E10	
**Number of molecules**	**Peak maximum**	**Photons/molecule**	**Peak maximum**	**Photons/molecule**
1	61.8	61.8	51.8	51.8
2	101.8	50.9	110.6	55.3
3	135.5	45.2	154.8	51.6
4	191.4	47.8	214.9	53.7
5	247.2	49.4	252.0	50.4
6	295.5	49.2	309.3	51.6
7	n/a	n/a	341.2	48.7
8	389.2	48.6	395.7	49.5
9	429.4	47.7	434.0	48.2
10	516.0	51.6	526.9	52.7
11	580.5	52.8	576.2	52.4
**Mean photons/molecule**		**51.0 ± 4.5**		**51.4 ± 2.13**

c

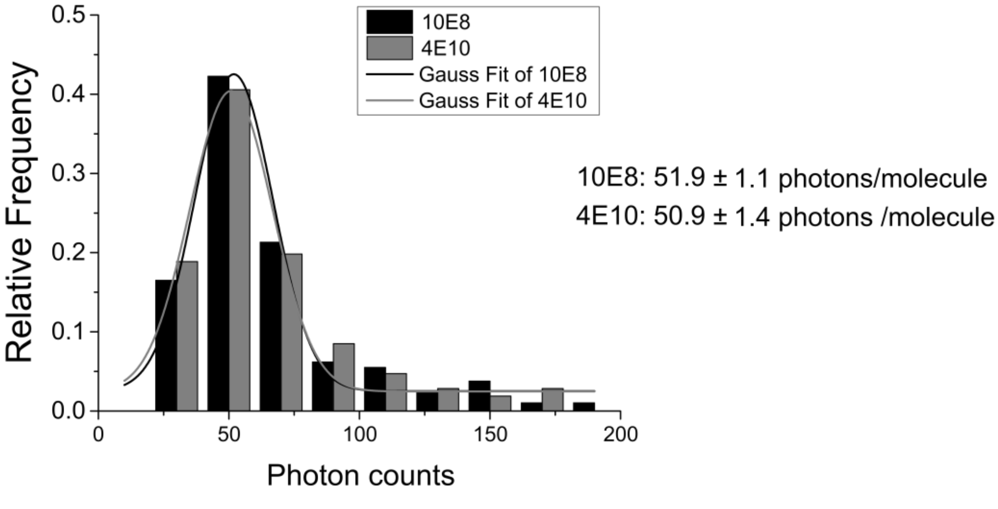

Supplementary Figure 3: Quantitation of photons emitted by KK114-labeled Fabs bound to JR-CSF virions. Frequency histograms (**a**) and calculated photons/molecule (mean values) after fitting frequencies to a normal distribution of the number of molecules (**b**). Photon/molecule values were the same as those obtained for Fabs stuck to the support in the absence of virions (**c**).

Supplementary Figure 4

a

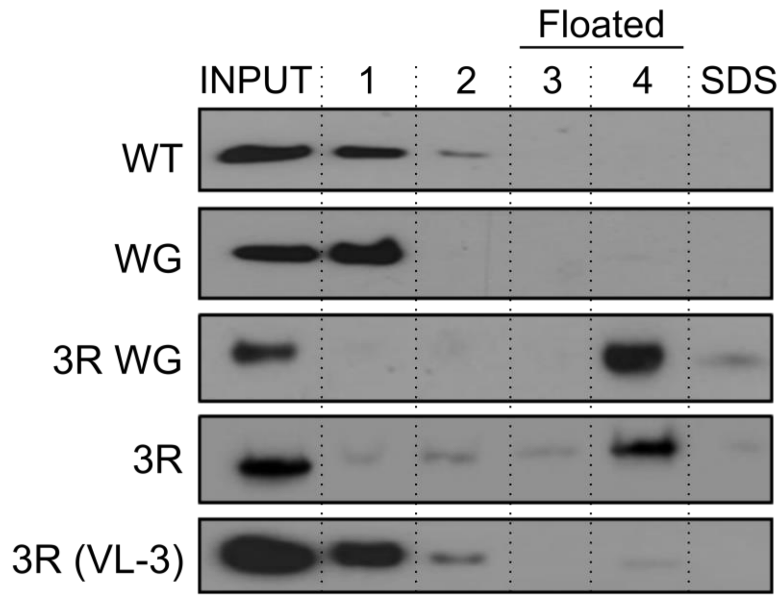

b

Fab Type	Mean (All)	Mean (Positive)	IC_{50} (µg/mL)
10E8	2.3 ± 0.2	4.3± 0.4	0.40 ± 0.08
10E8 3R	2.9 ± 0.3	5.4 ± 0.9	0.04 ± 0.01
10E8 3R WG	0.9 ± 0.1	1.8± 0.1	6.38 ± 0.71
10E8 WG	0.7 ±0.1	1.4 ±0.1	> 30

Supplementary Figure 4: Functional properties of 10E8 Fabs mutated in the MAPA/HCDR3 elements. (**a**) Membrane interaction (spontaneous partitioning) assessed by flotation of large unilamellar vesicles in a sucrose gradient (see [1] for a detailed description of this assay). In these assays the floated vesicles are found in fractions 3 and 4 and, therefore, Fab collected in these fractions is assumed to be membrane-bound (e.g., 3R mutants). Material recovered from the fractions 1 and 2 is assumed to be detached from vesicles. SDS is used to extract the Fab fraction unspecifically bound to the tube. Membrane composition of the vesicles (DOPC:DOPS 1:1, mole ratio), was selected to facilitate MAPA-mediated electrostatic interaction with membranes. VL-3 stands for "virus-like composition 3", which emulates the degree of lipid packing existing in the viral membrane (see [2] and caption for Supplementary Fig. S6 below). Fab detection was carried out through Western blot analysis of the different fractions. (**b**) Number of Fabs per virion (*F/v* values) determined by quantitative STED imaging, and neutralization activity of the different mutants measured against tier-2 JR-CSF pseudovirus (IC_{50} values).

Supplementary Figure 5

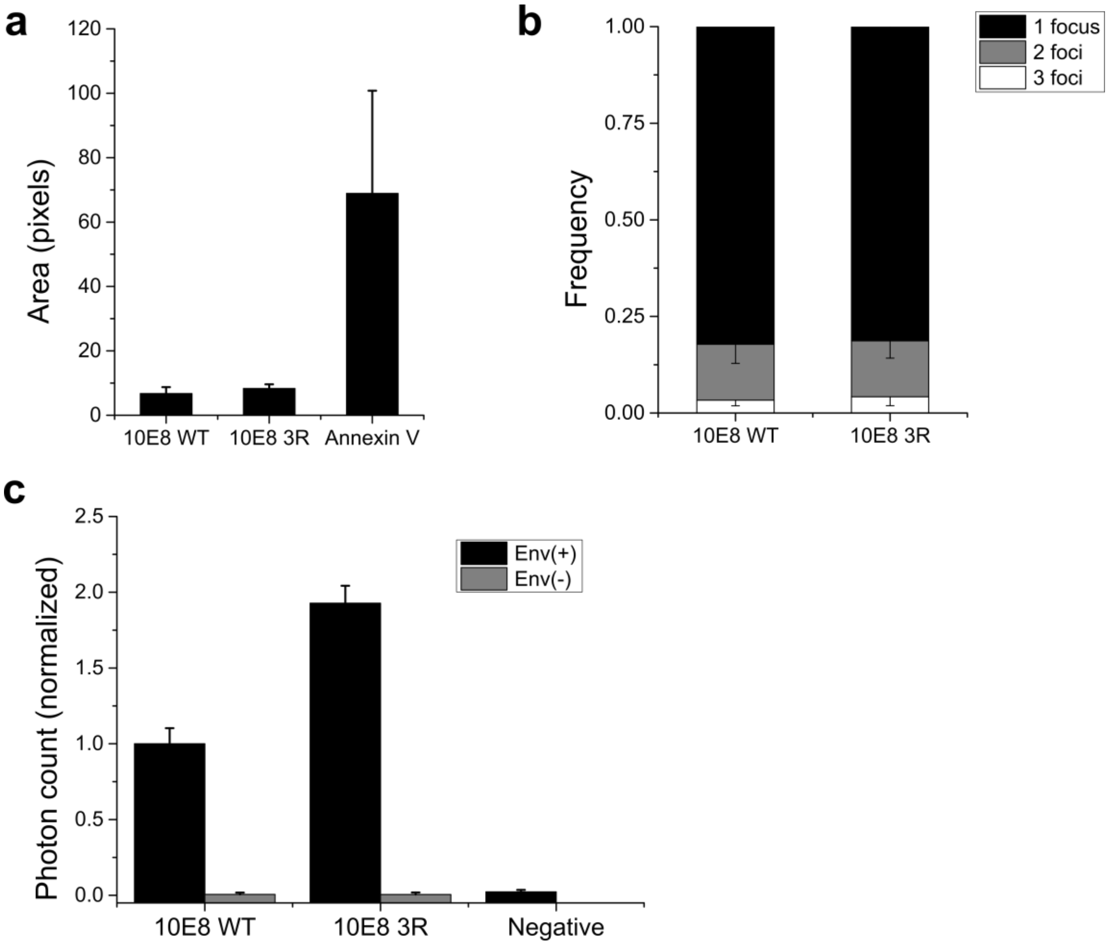

Supplementary Figure 5: Binding properties of 10E8 3R mutant established through STED microscopy in HIV-1 virions (NL4-3). (**a**) Mean pixel area of 10E8 and the 3R mutant Fab STED signals as compared to Annexin V-ATTO 647. (**b**) STED foci distribution in virions. (**c**) KK114-10E8-3R STED intensity signals in Env (+) and Env (-) virions (normalized to KK114-10E8-WT intensity and after background subtraction). In all panels, error bars are SD.

Supplementary Figure 6

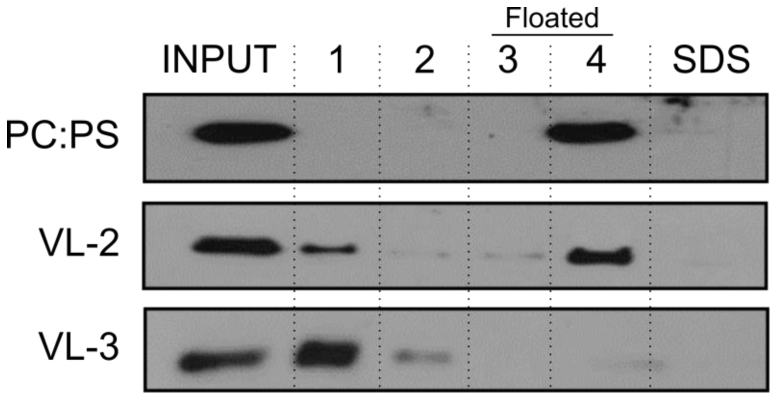

Supplementary Figure 6. Lipid composition dependence of Fab 4E10 interaction with membranes (spontaneous partitioning), as detected by flotation assays of large unilamellar vesicles in a sucrose gradient. Lipid compositions were: PC:PS: DOPC:DOPS 50:50; VL-2: DOPC:CHOL:ESM:DOPE:DOPS 14:46:17:16:7; VL-3: POPC:CHOL:ESM:POPE:POPS 14:46:17:16:7.

Supplementary Table 1. Neutralization ($IC_{50,}$ µg mL^{-1}) of JR-CSF viruses by unlabeled and KK114-labeled Fabs as measured in cell entry assays.

	**Unlabeled Fab**	**KK114-Fab**
10E8	0.31 ± 0.13	0.35 ± 0.18
4E10	4.58 ± 0.91	3.89 ± 0.58

Supplementary Table 2. Fabs/virion (*F/v*) values as calculated from the number of emitted photons in Vpr.eGFP positive ROIs, taking into account every viral particle (*All*) or only those positive for KK114 emission (*Positive*).

**HIV-1 Isolate**	**Antibody**	**[Fab] (ng µL^{-1})**	***F/v* (Mean) (All)**	***F/v* (Mean) (Positive)**	***F/v* (Median) (All)**	***F/v* (Median) (Positive)**
NL4-3 (Tier 1)	10E8	50	2.5 ± 0.1	3.5 ± 0.1	1.7	2.7
		100	4.4 ± 0.2	6.2 ± 0.2	2.9	4.7
	4E10	50	2.6 ± 0.1	3.9 ± 0.2	1.5	2.9
		100	4.4 ± 0.1	5.9 ± 0.2	3.0	4.7
JR-CSF (Tier 2)	10E8	50	2.3 ± 0.2	4.3 ± 0.4	1.1	2.4
		100	2.9 ± 0.2	5.4 ± 0.4	1.5	3.4
	4E10	50	1.6 ± 0.1	3.5 ± 0.3	0.8	2.2
		100	2.0 ± 0.1	3.7 ± 0.3	1.1	2.3

Supplementary Methods

Liposome flotation assays

Large unilamelar vesicles were prepared following the extrusion method. Lipids were mixed in chloroform and dried under a N_2 stream. Traces of organic solvent were removed by 2 h vacuum pumping. Subsequently, the dried lipid films were dispersed in buffer and subjected to 10 freeze-thaw cycles prior to extrusion 10 times through 2 stacked polycarbonate membranes with a nominal pore size of 100 nm (Nuclepore, Inc., Pleasanton, CA). Phospholipid concentration of liposome suspensions was determined by phosphate analysis. Vesicle flotation experiments in sucrose gradients were subsequently performed. In brief, 100 µL of a sample containing rhodamine-labeled liposomes and Fab (1.5 mM lipid and 1.5 µM Fab) was adjusted to a sucrose concentration of 1.4 M in a final volume of 300 µL and subsequently overlaid with 400 µL and 300 µL layers of 0.8 M and 0.5 M sucrose, respectively. The gradient was centrifuged at 436,000 ×g for 3 h in a TLA 120.2 rotor (Beckman Coulter, Brea, CA). After centrifugation, four 250 µL fractions were collected. The material adhered to the tubes was collected into a fifth fraction by washing with 250 µL of hot (100 ºC) 1% (w/v) SDS. The different fractions were run on SDS-PAGE, and the presence of Fab was probed by Western blotting using a goat (anti-human Fab) antibody (Sigma) and a mouse (anti-goat) antibody-HRP conjugate (Santa Cruz). The results displayed in the figures are representative of at least two replicates.

Supplementary References

1. Rujas, E. *et al.* Functional Optimization of Broadly Neutralizing Hiv-1 Antibody 10E8 By Promoting Membrane Interactions. *J. Virol.* JVI.02249-17 (2018). doi:10.1128/JVI.02249-17

2. Huarte, N. *et al.* Functional organization of the HIV lipid envelope. *Sci. Rep.* **6,** 34190 (2016).
